# Photoelectric and Self-Assembly Properties of Tetrasubstituted Pyrene Discotic Derivatives

**DOI:** 10.3390/molecules27217559

**Published:** 2022-11-04

**Authors:** Yuzhen Zhao, Yang Yu, Xiangrong Zhao, Yang Zhao, Zhun Guo, Huimin Zhang, Ruijuan Yao, Xinyu Ji, Dong Wang

**Affiliations:** 1 Xi’an Key Laboratory of Advanced Photo-Electronics Materials and Energy Conversion Device, School of Electronic Informat, Xijing University, Xi’an 710123, China; 2 Department of Materials Physics and Chemistry, School of Materials Science and Engineering, University of Science and Technology Beijing, Beijing 100083, China

**Keywords:** pyrene derivatives, self-assembly, nano/micro structures, π-conjugated structures, optoelectronic applications

## Abstract

To investigate the self-assembly behavior of π-conjugated ethynyl-pyrene discotic derivatives, a series of ethynyl-pyrene discotic materials were designed and synthesized by Sonogashira coupling reaction. The π-conjugated structures were characterized by ^1^H-NMR, IR spectroscopy, and elemental analysis. The optical properties of the discotic materials were examined by UV/Vis spectra and fluorescence emission spectra. The band gap of each compound was calculated by cyclic voltammetry with UV/Vis spectroscopy. Interestingly, the substituted groups in the four symmetrical positions did affect the self-assembly properties of as-resulted nano/micro structures. Under the same conditions, compounds **4a**–**4d** could be self-assembled into different morphologies such as micro-tubes (for **4a**), micro-wires (for **4b** and **4c**), and micro-grain crystals (for **4d**). All of the results indicated that the discotic materials have the potential for optoelectronic applications.

## 1. Introduction

In recent years, extended π-conjugated organic materials with excellent self-assembled microstructures have been increasingly applied in the field of organic optoelectronics [1,2,3,4,5,6,7], such as organic field-effect transistors (OFETs), organic light-emitting diodes (OLEDs), liquid crystal displays (LCDs), solar cells, and optical storage devices. As one of the important categories of expanding π-conjugated organic materials, discotic liquid crystal materials have attracted more and more attention owing to their excellent self-assembly and self-healing properties [8,9,10]. Besides, with the deepening of research, discotic organic liquid crystal has potential low cost and its unique properties can be customized by chemical reaction design [11,12], which gives it broad development prospects.

Pyrene molecules with a large conjugated structure, good fluorescence properties, and chemical stability are one of the ideal structures for self-assembled discotic liquid crystal molecules [13,14,15]. Organic molecules with pyrene as the core are widely used in the field of probes because of their high fluorescence efficiency and excimer formation [16,17,18]. Many pyrene derivatives also show liquid crystal (LC) behavior [19]. Recently, the self-assembly behaviors of pyrene core have often been studied to promote the mobility of organic field-effect transistors. For example, 1D single-crystalline building blocks from π-conjugated pyrene molecules for applications in organic field-effect transistors with relatively high mobility have been reported [20]. Owing to its well-defined single-crystalline microstructures grown by strong π–π interactions, the performance is outstanding.

In this work, a series of pyrene derivatives with different π-conjugated lengths and donor groups were designed and synthesized by Sonogashira coupling reaction. The structures of pyrene, alkyne, and benzene ring enlarge the conjugated structure of molecule and increase the π–π stacking effect, and the grafting of alkyl chain improves the solubility of molecule and neutralizes the steric hindrance effect of the side chain. The self-assembly behavior and photoelectric properties of pyrene derivatives with different π-conjugated degrees were studied, and the results were also consistent with the expectations. This provides a certain reference for self-assembled organic photoelectric disk-mounted molecules, and the organic photoelectric molecules will certainly exert greater value and contribution in the future.

## 2. Results and Discussion

**Scheme 1:**
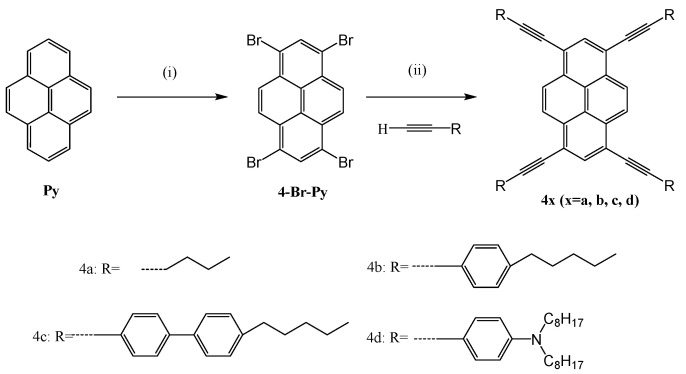
Synthesis of 1,3,6,8-tetrabromopyrene (4-Br-Py) and 1,3,6,8-tetrakis(ethynyl)-pyrene derivatives 4a–4d. Reagents and conditions: (i) Br_2_, Ph-NO_2_, 130 °C, for 12 h, 80%. (ii) PdCl_2_(PPh_3_)_2_, CuI, PPh_3_, Et_3_N/THF (1:1), 12 h, 80 °C.

Scheme 1 shows the chemical structures of tetrakis(arylethynyl) pyrenes **4a**–**4d** used in this study. The tetrakis(arylethynyl) pyrenes **4a**–**4d** were prepared by Sonogashira coupling reaction of tetrabromopyrene with the corresponding arylacetylenes. These compounds consist of pyrene, acetylene, and aryl/moieties. Here, the aryl moieties include electron-donating R substituents at the para position against the acetylene one. In [21], the R may be an electron-donating, H, or electron-withdrawing group. Pyrene itself is electrically neutral, thus its electric characteristic will depend on those of the R substituents through π-conjugation. Therefore, when the R is an electron-donating group, the pyrene moiety is envisaged to behave as an acceptor, and vice versa. In this paper, the effects of substituents on the self-assembly and optical properties were discussed in detail.

### 2.1. Spectroscopic Characterizations

**Figure 1:**
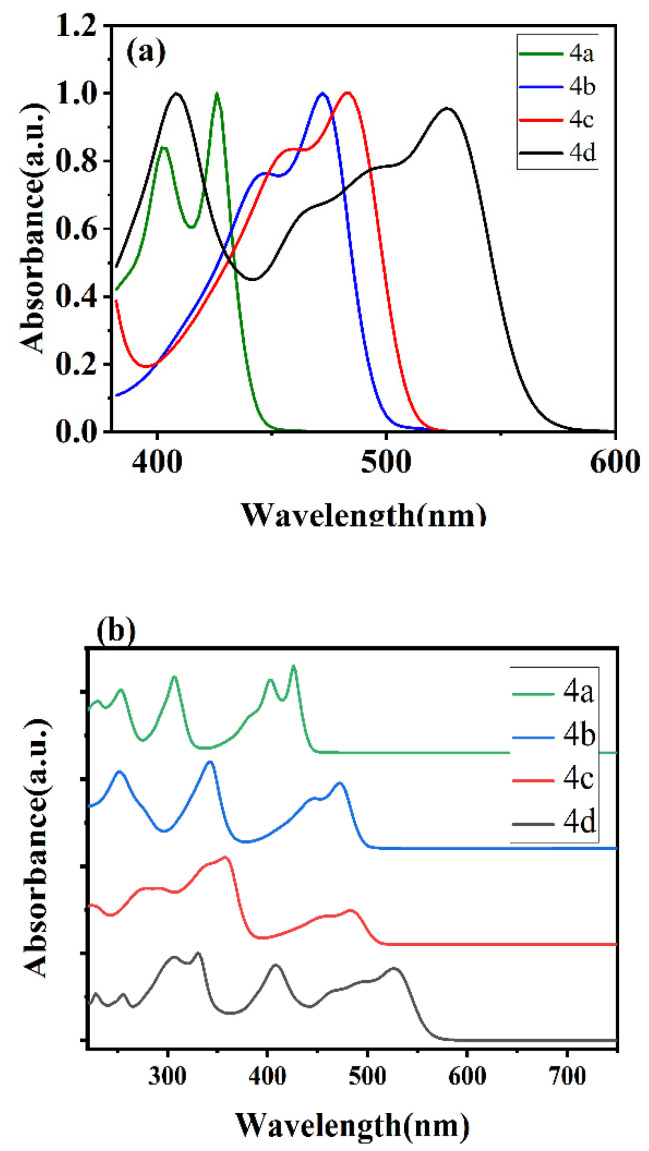

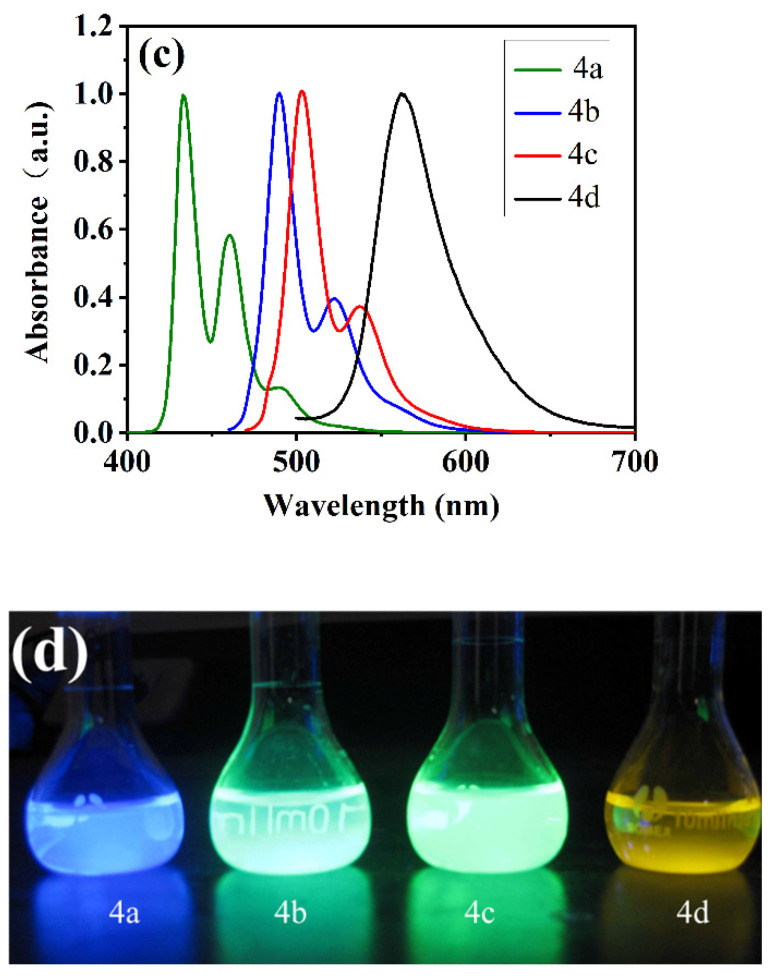
(**a**) UV/Vis spectra of **4a**–**4c** in CH_2_Cl_2_: [4a–4d] = 1.0 × 10^−4^ M; (**b**) normalized UV/Vis spectra of **4a**–**4c** in CH_2_Cl_2_: [**4a**–**4d**] = 1.0 × 10^−4^ M; (**c**) normalized-fluorescence spectra of **4a**–**4d** in CH_2_Cl_2_: [**4a**–**4d**] = 1.0 × 10^−6^ M were excited at the each λ_abs_^max^ wavelength; (**d**) the photos of **4a**–**4d** in CH_2_Cl_2_: [**4a**–**4d**] = 1.0 × 10^−6^ M were excited at 254 nm.

All compounds show complex absorption spectra. The absorption spectra of compound **4a** in diluted CH_2_Cl_2_ solutions show two main absorption band peaks at 426 and 402 nm (Figure 1a,b and Table 1). The absorption peaks at 306 nm and 253 nm may be related to the electron transfer caused by the alkyl chain (Figure 1b). The absorption spectrum of compound **4b** is similar, but shows a pronounced bathochromic shift of the main absorption band peaks now located at 472 and 446 nm (Figure 1a,b and Table 1). The 44 nm bathochromic shift of the absorption peak was attributed to the elongation of conjugation. Compared with **4b**, **4c** shows a slight bathochromic shift of the main absorption band peaks located at 483 and 457 nm (Figure 1a,b and Table 1). The reason can also be attributed to the elongation of conjugation. The maximum absorption peak of **4d** was observed at 526 nm bathochromically shifted by ca. 43 nm as compared with **4c**. Given the π-conjugation extension from **4a** to **4c**, it was shown that the bathochromic shift between **4c** and **4d** is most likely due to the electron supplying capacity of donor substituents.

Compared with more than four peaks in the UV spectra, only one or two peaks were found in the fluorescence spectra of **4a**–**4d**, and the results are shown in Figure 1c. However, the use of pyrenes as emitters in OLEDs is limited because pyrenes easily form π aggregates/excimers in concentrated solution and the solid state, and the formation of π aggregates/excimers leads to long-wavelength excimer emission with low quantum efficiency [22]. Exciting the low energy absorption bands of **4a** leads to strong emissions at 432 nm and 460 nm with a quantum yield of 21% (Table 1). Exciting the low energy absorption bands of **4b**, the emission is 490 nm and 522 nm, red-shifted by 60 nm as compared with compound **4a** with a quantum yield of 41% (Table 1). In contrast with **4a** to **4c**, for **4d**, only one emission band was observed at 562 nm, indicating that the emission occurred in the lowest excited state with the maximum oscillator strength. Upon excitation, a dilute solution (ca. 10^−6^ M) of **4a**–**4d** in CH_2_Cl_2_ at room temperature showed blue, green, and yellow emission, respectively (Figure 1d). For example, compared with the lowest-energy emission band of pyrene **4d** at 562 nm, the lowest-energy emission band of **4a**, **4b**, and **4c** occurred at 460, 522, and 537 nm, respectively. The fluorescence Stokes shifts (in Table 1) increased in the order of **4d** < **4a** < **4b** < **4c**.

### 2.2. Electrochemical Properties

**Figure 2:**
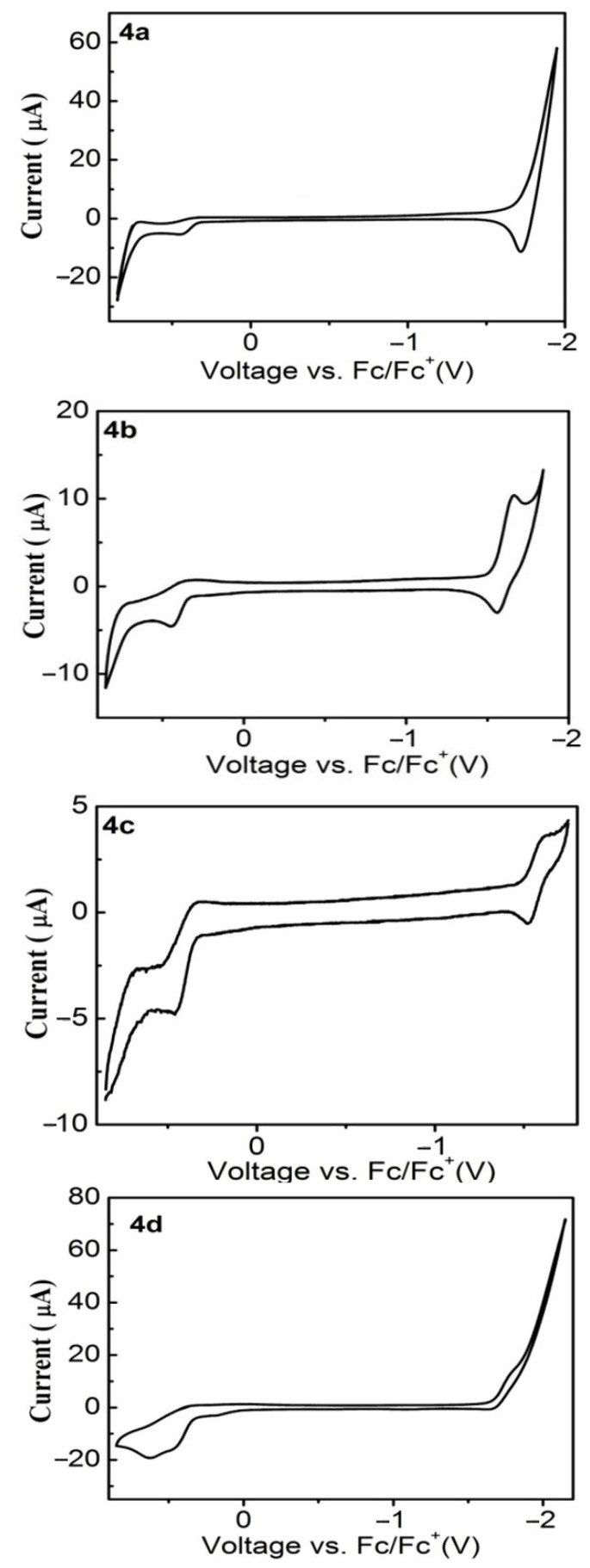
Cyclic voltammogram of the compounds.

All compounds were characterized by cyclic voltammograms, as shown in Figure 2, and the data are listed in Table 2. The irreversible oxidation potential was observed in CH_2_Cl_2_ for **4d**. For **4a**–**4c**, the reversible oxidation potentials were observed as shown in Figure 2.

The onset oxidation potentials for **4a**–**4d** were located at 0.34, 0.35, 0.36, and 0.06 V, respectively. According to the equation HOMO = −(E_ox_^onset^ + 4.8 − 0.2) eV, their respective HOMO energy levels were estimated to be −4.94, −4.95, −4.96, and −4.66 eV, respectively. These values are similar to those of N,N′-di(1-naphthyl)-N,N′-diphenylbenzidine (NPB, 5.46 eV), which is one of the most widely used hole-transport materials. Thus, they would be beneficial for hole injection and transportation.

Subsequently, the onset reduction potentials and LUMO energy levels for **4a**–**4d** were estimated and are shown in Table 2. The optical band gaps were estimated to be 2.27, 2.02, 1.98, and 1.73 eV. The optical band gaps were obtained from the edge absorption of the diluted CH_2_Cl_2_ solution samples. Furthermore, these electrochemical band gaps were in good correlation with the optical spectroscopic (optical band gaps).

Consequently, the LUMO, HOMO, and band gaps were gradually lowered owing to the increase in π electron numbers and donor groups. In this system, there is the smaller effect of conjugation length on the electrochemical properties than that of donor groups. This can be mainly attributed to the strong charge-transfer character of donor groups in these typical conjugation compounds.

### 2.3. Self-Assembly of Pyrene Derivatives

**Figure 3:**
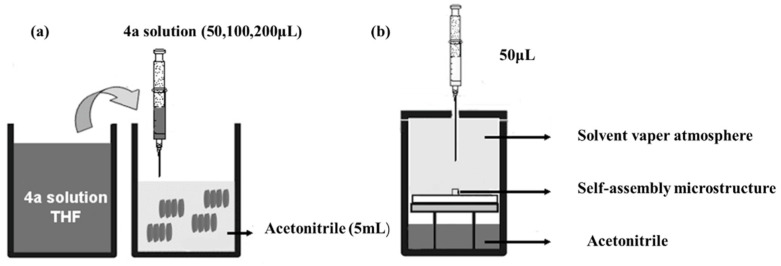
Fabrication steps for the preparation of **4a**–**4d** microstructures. (**a**) The first step: the compounds through the solvent-exchange method in the solution phase, which transfers the molecule from a good solvent (THF) into a poor solvent (acetonitrile, 5 mL); (**b**) the second step: the solution dropping to a SiO_2_ substrate. The solvent is allowed to evaporate slowly or quickly in a closed jar, giving rise to different microstructures.

The translation of well-designed pyrene derivatives into nano- and microscopic objects such as nanowires, nanoribbons, and nanobelts has attracted significant interest for potential applications in electronics and optoelectronics [23,24,25]. For organic micro/nanomaterials, solution processes have been widely applied in devices fabrication owing to the convenience and low cost. Figure 3 shows the self-assembly method used in this work. At first, we studied the self-assembly behaviors of compound **4a**.

**Figure 4:**
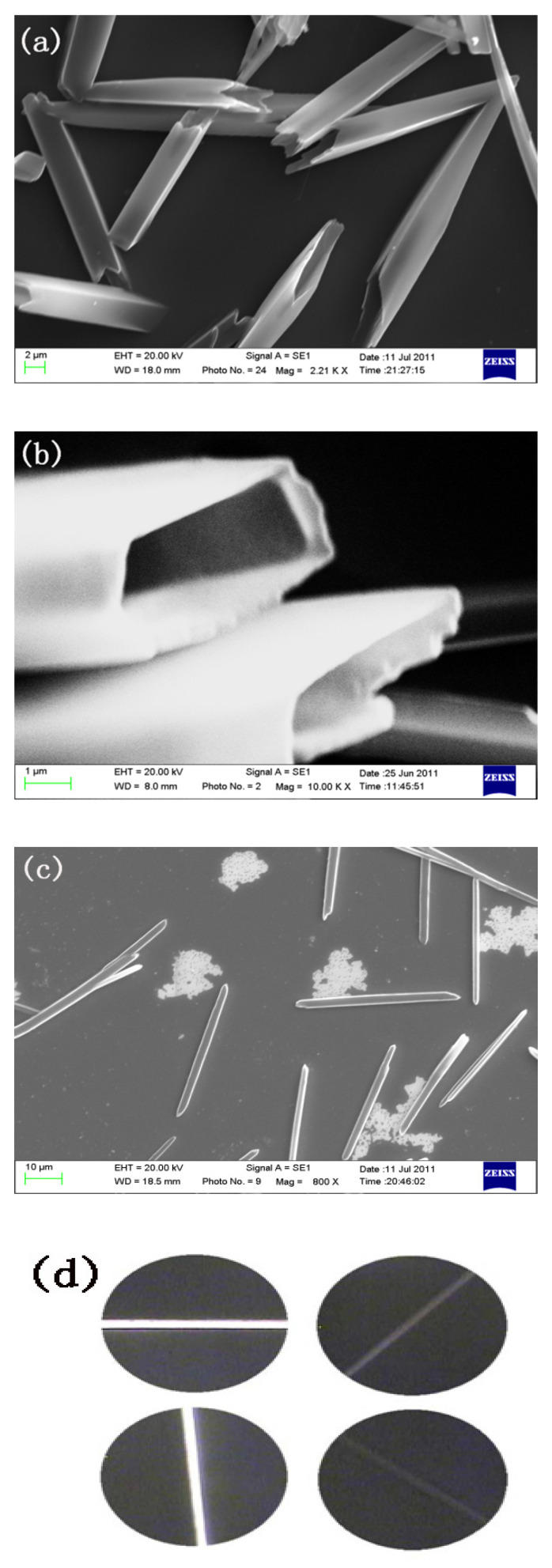
(**a**,**b**) SEM of micro-square tube by **4a** self-assembly; (**c**) SEM of acicular crystal by **4a** self-assembly; (**d**) POM of micro-square tube by **4a** self-assembly with different angles.

Figure 4 illuminates the SEM images of compound **4a**. The micro square tubes and microcuboids were prepared from **4a** powder as the starting material using the solvent-exchange method in the solution phase. The conformational flexibility of the alkyl side group of the pyrene derivative **4a** molecule gives it sufficient solubility in THF, and the conjugated structure of pyrene and alkyne promotes the π–π stacking effect, thus the material shows the self-assembly effect on the macro level [26,27].

The **4a** molecule becomes insoluble in special solvents such as methanol, ethanol, and acetonitrile. As shown in Figure 3a, the injection of a minimum volume of concentrated THF solution of pyrene derivative **4a** into acetonitrile led to the formation of nanocrystals through self-assembly and the growth of a microstructure in the closed chamber (Figure 3b).

From the results, to obtain a high-quality micro-square tube, one of the key factors is to inhibit the fast evaporation of the solvent in the **4a** solution. If the jar is not filled with acetonitrile, the vapor pressure of the solvent will remain low in the closed jar. At this time, the evaporation of **4a** solution will be relatively fast, creating conditions for the manufacture of the micro-square tube structure.

Figure 4a shows that the micro-square tube grew out of the jar and its solvent evaporated in a short time (room temperature, within 1 min). Before adding the 4a solution dropwise to the substrate, acetonitrile (10 mL) was added dropwise to the jar in order to maintain a high solvent vapor pressure. Based on this, the evaporation of the **4a** solution will be relatively slow, which promotes molecular self-assembly to form the microstructures (micro-cuboids) in Figure 4c. The results that emerged from the experiments may be due to the interaction of the self-assembly of the pyrene disk structure with the spatial structure of its substituents [14,20,28]. However, from the POM results in Figure 4d, both of the microstructures show the typical characteristic of single crystal materials. The alternating dark and bright with the different angles was observed, and it can be proved that the pyrene discotic molecules were regularly arranged in the microstructures. This is similar to the results obtained in the previous literature [24,25,26].

**Figure 5:**
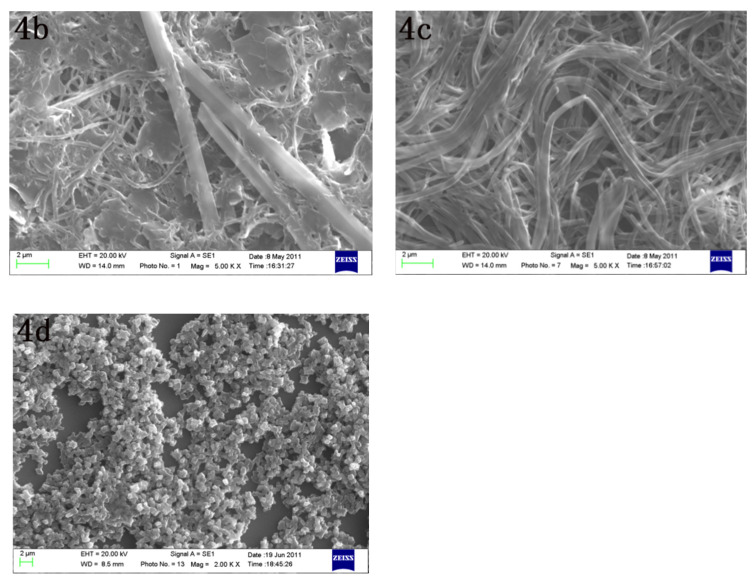
SEM images of micro-square tube by **4b**–**4d** self-assembly.

We also use the same method to study the other compounds; the other types of self-assembly morphology were observed from SEM and the results are shown in Figure 5. For **4b** and **4c**, the micro-wire morphology was fabricated by the same method as for **4a**. The long microstructures can contribute to the stronger short range intermolecular interaction. For **4d**, the intramolecular interaction is obviously weaker, so the self-assembly structures of **4d** showed smaller sizes than those of others. However, the micro-square tube cannot be observed for **4b**–**4d**.

## 3. Experimental Section

### Materials and Methods

All of the reagents were purchased as reagent grade from commercial sources (Aldrich, St Louis, MO, USA) and used without further purification. Triethylamine (TEA, Et_3_N) and tetrahydrofuran (THF) were distilled and purged with argon before use. A Bruker DMS-400 spectrometer (Brucker, Karlsruhe, Germany) was used to record the ^1^H NMR spectra at 25 °C. CDCl_3_ was the solvent for NMR and chemical shifts relative to tetramethyl silane (TMS) at 0.00 ppm are reported in parts per million (ppm) on the d scale. The resonance multiplicity was described as s (singlet), d (doublet), and m (multiplet). MALDI-TOF negative ionization mass spectra were recorded on a Shimadzu spectrometer (Shimadzu, Kyoto, Japan), using dithranol as the matrix. Elemental analyses were performed at the Institute of Chemistry Chinese Academy of Sciences, with a Flash EA 1112 instrument (ThermoFinnigan, San Jose, CA, USA). All UV/Visible spectra were recorded on a JASCO V-570 spectrophotometer (JASCO, Tokyo, Japan). FT-IR spectroscopy was recorded on a Perkin Elmer LR-64912C spectrophotometer (Perkin Elmer, Waltham, MA, USA) and all fluorescence spectra were recorded on a HITACHI F-4500 fluorescence spectrophotometer (HITACHI, Tokyo, Japan). Differential scanning calorimetry (DSC) analyses were performed on a Perkin Elmer Pyris 6 instrument (Perkin ElmerWaltham, MA, USA). Scanning electron microscopy (SEM) observation was performed with a JEOL JSM-5400/LV (JEOL, Tokyo, Japan). The accelerating voltage was 15 kV. The optical textures of the compounds were observed via a polarized optical microscope (Nikon, Tokyo, Japan) equipped with a hot stage calibrated to an accuracy of ±0.1 K (Linkam LK-600PM, Linkam, Salfords, UK). The redox properties of PT, PTE, and PTQ were investigated by cyclic voltammetry (CV) in CH_2_Cl_2_ (1 × 10^−5^ M, 0.1 MnBu_4_NPF_6_, all potentials versus the ferricinium/ferrocene couple (Fc+/Fc)). Cyclic voltammetric measurements were carried out in a conventional three-electrode cell using glassy carbon working electrodes of 2 mm in diameter, a platinum wire counter electrode, and a Ag/AgCl reference electrode on a computer-controlled CHI 660C instrument at room temperature (Chenhua Instrument, Shanghai, China). The energy levels were calculated using the Ferrocene (Fc) value of −4.8 eV with respect to the vacuum level, which was defined as zero. The measured oxidation potential of Fc (vs. Ag/AgCl) was 0.20 V. Therefore, the HOMO energy (E_HOMO_) levels of the products could be calculated by the equation E_HOMO_ = −[E_onset_^ox^ − E_1/2_, _Fc_ + 4.8] eV, and the LUMO energy (E_LUMO_) levels could be estimated by the equation E_LUMO_ = −[E_onset_^red^ − E_1/2_, _Fc_ + 4.8] eV, where E_1/2_, _Fc_ stands for the half-wave potential of Fc/Fc^+^ [28].

1,3,6,8-tetrabromopyrene: The compound was synthesized based on the method in the [29].

1,3,6,8-tetra(hex-1-ynyl)pyrene (**4a**): A degassed THF/Et3N (20/20 mL) mixed solution of 1,3,6,8-tetra-bromopyrene (0.51 g, 1.0 mmol), PdCl_2_(PPh_3_)_2_ (70.4 mg, 0.1 mmol), CuI (38.0 mg, 0.2 mmol), PPh_3_ (26.2 mg, 0.1 mmol), and hex-1-yne (0.49 g, 6.0 mmol) was refluxed under argon protection for 8 h. The reaction mixture was then cooled to room temperature and filtered. The filtrate was evaporated and the residue was purified by column chromatography (silica gel, 2:8 CH_2_Cl_2_/hexane) to give the following (green powder): yield 49% (0.238 g) mp: 107 °C. ^1^H NMR (CDCl_3_, TMS, 400 MHz) δ = 8.54 (s, 4H), 8.14(s, 2H), 2.64 (t, J = 6.8 Hz, 8H) 1.74 (m, J = 7.2 Hz, 8H), 1.61 (m, J = 7.2 Hz, 8H), 1.03 (t, J = 7.2 Hz, 12H). IR ν (cm^−1^): 2954, 2930, 2864, 2225, 1775, 1601, 1509, 1465, 1435, 1385, 1323, 1245, 1106, 1009, 890, 823, 714, 631, 534. MALDI-TOF-MS (dithranol): *m*/*z*: calcd for C40H42: 522.33 g·mol^−1^, found: 523.4 g·mol^−1^ [MH]+. Elemental analysis calcd (%): C 91.90, H 8.10; found: C 91.89, H 8.11.

1,3,6,8-tetrakis((4-pentylphenyl)ethynyl)pyrene (**4b**): The reaction mode is consistent with the synthesis method of 4a. Under the same conditions, the reaction product was changed from hex-1-yne (0.49 g, 6.0 mmol) to 1-ethynyl-4-pentyl Benzene (1.03 g, 6.00 mmol). The reaction mixture was then cooled to room temperature and filtered. The filtrate was evaporated and the residue was purified by column chromatography (silica gel, 1:9 CH_2_Cl_2_/hexane) to give the following (golden powder): yield 54% (0.477 g) mp: 147 °C. ^1^H NMR (CDCl_3_, TMS, 400 MHz) δ = 8.66 (s, 4 H), 8.37 (s, 2H), 7.63 (d, J = 8.0 Hz, 8H), 7.24 (d, J = 8.0 Hz, 8H), 2.67 (t, 8H), 1.67 (m, 8H), 1.36 (m, 16H), 0.93 (t, 12H). IR ν (cm^−1^): 3034, 2957, 2924, 2853, 2193, 2140, 1907, 1784, 1597, 1506, 1461, 1416, 1370, 1286, 1176, 1111, 1014, 904, 832, 722, 554. MALDI-TOF-MS (dithranol): *m*/*z*: calcd for C68H66: 882.52 g·mol^−1^, found: 883.5 g·mol^−1^ [MH]+. Elemental analysis calcd (%): C 92.47, H 7.53; found: C 92.47, H 7.53.

1,3,6,8-tetrakis((4-pentylbiphenyl-4-yl)ethynyl)pyrene (**4c**): The reaction mode is consistent with the synthesis method of 4a. Under the same conditions, the reaction product was changed from hex-1-yne (0.49 g, 6.0 mmol) to 4-ethynyl-4-pentylbiphenyl (1.49 g, 6.00 mmol). The reaction mixture was then cooled to room temperature and filtered. The filtrate was evaporated and the residue was purified by column chromatography (silica gel, 1:9 CH_2_Cl_2_/hexane) to give the following (red powder): yield 48% (0.570 g) mp: 197 °C. ^1^H NMR (CDCl_3_, TMS, 400 MHz) δ 8.70 (s, 4H), 8.43 (s, 2H), 7.65 (d, J = 7.6 Hz,8H), 7.59 (m, J = 8.8 Hz, 8H), 7.56 (d, J = 8.6 Hz, 8H), 7.51 (d, J = 8.4 Hz, 8H), 2.66 (m, J = 8.6 Hz, 8H), 1.67 (m, 8H), 1.37 (m, 16H), 0.92 (t, J = 7.2 Hz, 8H). IR ν (cm^−1^): 3027, 2957, 2925, 2847, 2348, 2200, 1907, 1785, 1599, 1500, 1396, 1292, 1241, 1183, 1117, 1072, 1001, 898, 832, 807, 735, 547, 509. MALDI-TOF-MS (dithranol): *m*/*z*: calcd for C92H82: 1186.64 g·mol^−1^, found: 1187.8 g·mol^−1^ [MH]+. Elemental analysis calcd (%): C 93.04, H 6.96; found: C 93.03, H 6.97.

4,4,4,4(pyrene1,3,6,8tetrayltetrakis(ethyne2,1diyl))tetrakis(N,N-dioctylaniline) (**4d**): The reaction mode is consistent with the synthesis method of 4a. Under the same con-ditions, the reaction product was changed from hex-1-yne (0.49 g, 6.0 mmol) to 4-ethynyl-N,N-dioctylaniline (2.05 g, 6.00 mmol). The reaction mixture was then cooled to room temperature and filtered. The filtrate was evaporated and the residue was purified by column chromatography (silica gel; 1:9 CH_2_Cl_2_/hexane) to give the following (dark red powder): yield 52% (0.811 g), mp: 120 °C. ^1^H NMR (CDCl_3_, TMS, 400 MHz) δ = 8.68 (s, 4H), 8.32 (s, 2H), 7.54 (d, J = 8.0 Hz, 8H), 6.64 (d, J = 8.0 Hz, 8H), 3.30 (t, 16H), 1.61 (m, 16H), 1.33 (m, 80H), 0.89 (t, 24H). IR ν (cm^−1^): 3093, 3041, 2924, 2847, 2270, 2199, 1687, 1610, 1526, 1467, 1416, 1370, 1299, 1267, 1228, 1189, 1137, 1066, 1002, 977, 933, 892, 832, 808, 720, 597, 561, 523, 489. MALDI-TOF-MS (dithranol): *m*/*z*: calcd for C112H158N4: 1559.25 g·mol^−1^, found: 1556.1 g·mol^−1^ [MH]+. Elemental analysis calcd (%): C 86.20, H 10.21, N 3.59; found: C 86.18, H 10.22, N 3.60.

## 4. Conclusions

In summary, a series of tetrasubstituted pyrene discotic materials with different conjugated lengths and cloud density substituted groups were prepared and characterized. The results obtained by UV/Vis spectra and fluorescence spectra of these compounds indicated the efficient intramolecular charge-transfer (CT) interactions and elongation of conjugation, which were the result of the enlargement of the absorption spectrum and the corresponding strongly bathochromically shifted CT bands. By self-assembly of pyrene derivatives with the different methods, different morphologies were observed by SEM, such as the micro-square tubes, micro-wires, and micro-grains. This result reflected the artificial control of microstructures via versatile methods, which might represent a substantial advancement in small-scale device applications.

## Figures and Tables

**Table 1:** Optical properties of products.

Compd	Absorption ^a^ λ_abs_ (nm)	Fluorescence ^b^ λ_em_ (nm)	Stokes Shift (cm^−1^)	Φf ^c^
**4a**	253, 306,402, 426 ^d^	432, 460	326	0.21
**4b**	252, 342,446, 472 ^d^	490, 522	778	0.41
**4c**	280, 350, 457, 483 ^d^	503, 537	823	0.39
**4d**	256, 306, 331, 408, 526 ^d^	562	1218	0.15

^a^ [**4a**–**4d**] = 1.0 × 10^−4^ M. ^b^ [**4a**–**4d**] = 1.0 × 10^−6^ M. ^c^ Fluorescence quantum yields, determined by fluorescein (measured in CH_2_Cl_2_ solution using quinine sulfate as a standard at room temperature). ^d^ Excitation wavelength.

**Table 2:** Electrochemical properties of the compounds.

Compd	E_g_^opt^ [eV] ^a^	E_g_^elec^ [eV]	E_ox_^onset^ [V]	HOMO [eV]	E_red_^onset^ [V]	LUMO [eV]
**4a**	2.75	2.03	0.34	−4.94	−1.69	−2.91
**4b**	2.48	1.87	0.35	−4.95	−1.52	−3.08
**4c**	2.33	1.85	0.36	−4.96	−1.50	−3.10
**4d**	2.13	1.73	0.06	−4.66	−1.67	−2.93

^a^ Band gap estimated from the cutoff wavelength of optical absorption in CH_2_Cl_2_ solution.

## Data Availability

Not applicable.
